# The Endodontic Treatment of Three-Rooted Maxillary Premolar Resembling a Small Molar: A Case Report

**DOI:** 10.7759/cureus.73634

**Published:** 2024-11-13

**Authors:** Rucha Pawar, Anand Bansod, Roshan M Samuel, Priyanka Zinge

**Affiliations:** 1 Department of Conservative Dentistry and Endodontics, School of Dental Sciences, Krishna Vishwa Vidyapeeth (Deemed to be University), Karad, IND

**Keywords:** endodontic failure prevention, endodontic therapy challenges, maxillary first premolar, missed canals, pulp chamber anatomy, three-rooted premolar, tooth morphology variation

## Abstract

An appropriate access cavity, sufficient cleaning, proper shape, and full obturation are necessary for a root canal to be successful. In the earliest stages of therapy, the location of all root canals within the tooth is crucial. Even if the morphology of most teeth is normal, some variations should be acknowledged. As a transitional tooth between incisors and molars, the maxillary first bicuspid is most frequently two-rooted, while it can also occasionally show as a three-rooted system. While it often has two canals, it can occasionally have three, and it is quite easy to overlook the third canal.

## Introduction

One of the important components of endodontic therapy includes a precise interpretation of preoperative radiographs and a complete grasp of the architecture of the root canal anatomy. Since missing canals are a common cause of endodontic failure, practitioners should be aware of all possible changes in each tooth's root canal system [1]. When a dentist misses a root canal, it is addressed without treatment, especially in teeth with many root canals [2,3]. The maxillary first bicuspid has two cusps, the buccal cusp being noticeably bigger than the palatal cusp. It has been reported that this tooth has the most diversity in root canal morphology and root anatomy [3,4]. From 0% to 6% of maxillary first bicuspid has three roots, and each root typically has one canal [5]. The mesiobuccal, distobuccal, and palatal canal anatomy of a maxillary premolar are comparable to those of the surrounding molars [4]. This report's objective is to examine a tooth involving three root canals in the right maxillary first premolar that underwent root canal treatment at the School of Dental Sciences, Krishna Vishwa Vidyapeeth (KVV), Karad.

## Case presentation

**Figure 1:**
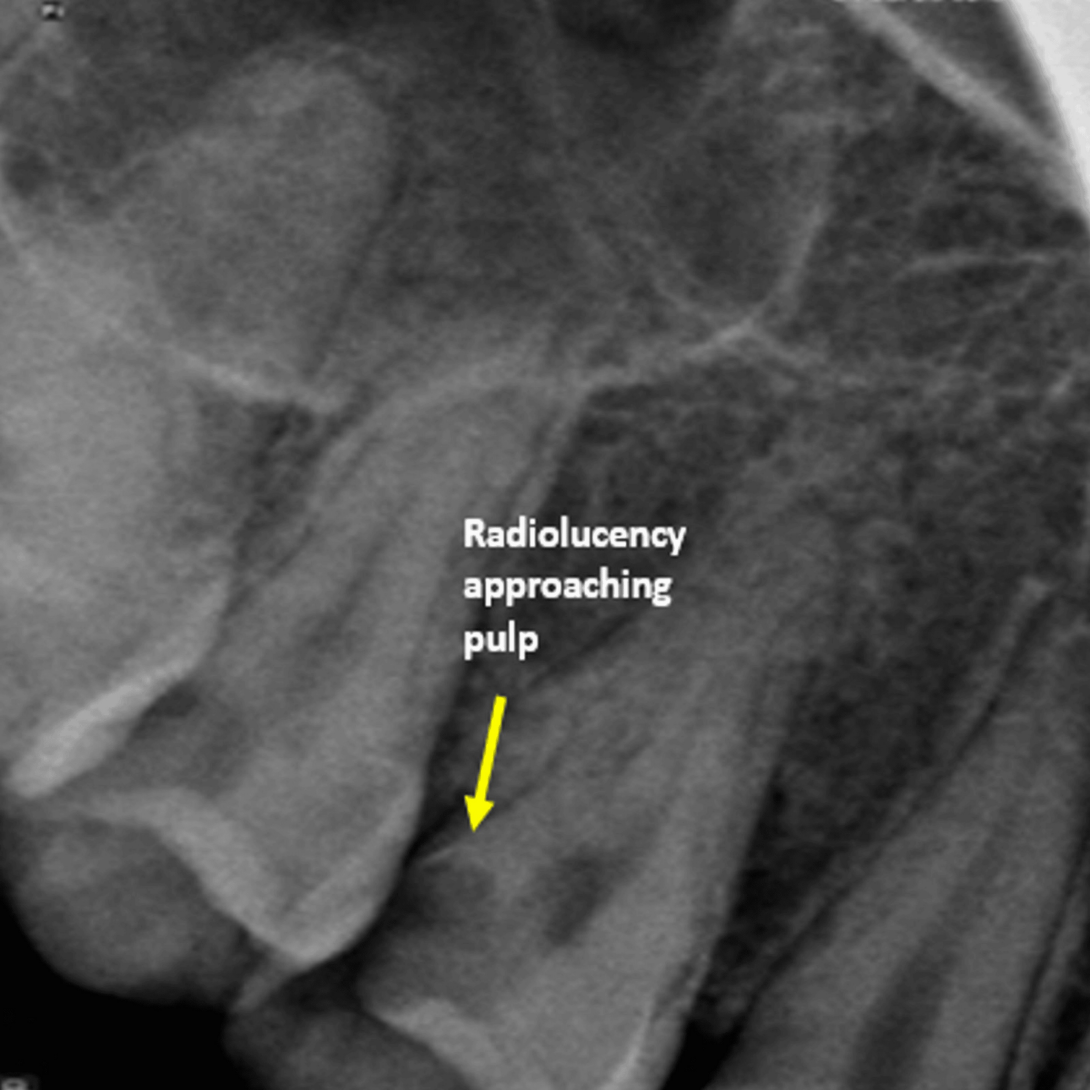
Preoperative radiograph

The primary complaint of a 22-year-old female patient who was seen at the Department of Conservative Dentistry and Endodontics, School of Dental Sciences, KVV, Karad, was pain in the upper right rear area of the jaw. The pain was dull and continuous in nature and was relieved after taking medication. The patient also gave a history of night pain and also symptomatic tenderness of percussion. On radiographic examination, there is radiolucency on the distal aspect of the tooth, which was approaching to pulp (Figure 1).

The widening of the periodontal ligament is seen associated with the tooth. A vitality test was done using an electric pulp test (EPT), which showed a delayed response to the tooth. After clinical and radiographic examination, we came to our diagnosis, symptomatic irreversible pulpitis with apical periodontitis. During the radiographic examination, we see unusual anatomy with the tooth; three outlines of roots are seen giving the idea that the tooth has three roots and three root canal systems.

**Figure 2:**
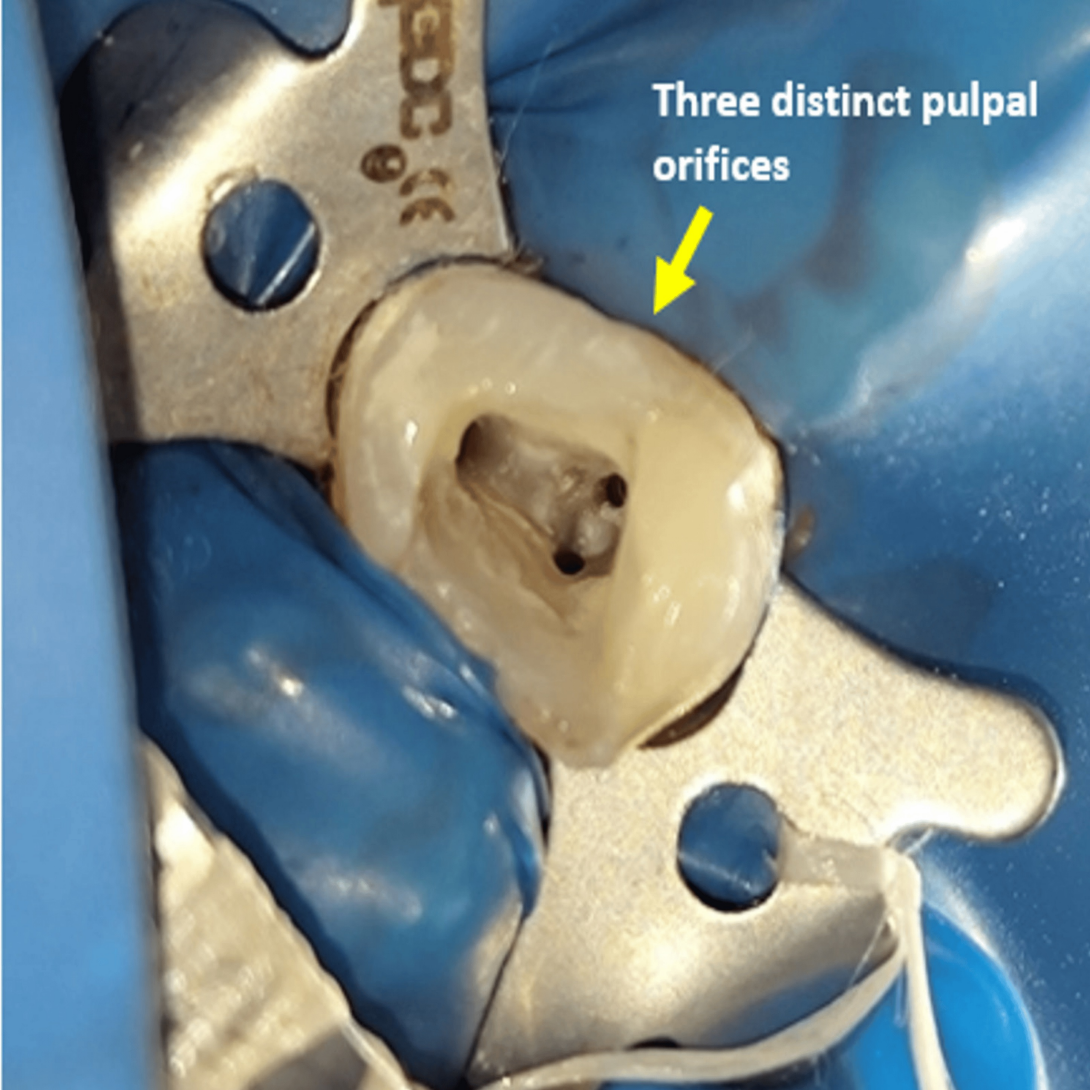
Clinical picture showing three different orifices

Before the implementation of the anesthetic, a test dose of 1:10 dilution of 2% lignocaine was executed intradermally on the forearm to determine whether the patient was allergic to local anesthetic. After confirming the patient's lack of anesthesia allergy, the root canal treatment was started. The patient was anesthetized with a solution of 2% lignocaine (Miracalus Pharma Ltd., Mumbai, India) with 1:100,000 epinephrine. The isolation of the tooth was done using a rubber dam (GDC Dental Rubber Dam Kit, Hoshiarpur, India). An occlusal reduction was performed, and coronal access was made using a sterile round bur (Mani Diamond Bur, Utsunomiya, Japan). After the excavation of the carious part, the pre-endodontic buildup was done (3M ESPE Filtek Z250 XT Restorative Kit, Seefeld, Germany), by which three distinct pulpal orifices were appreciated (Figure 2).

**Figure 3:**
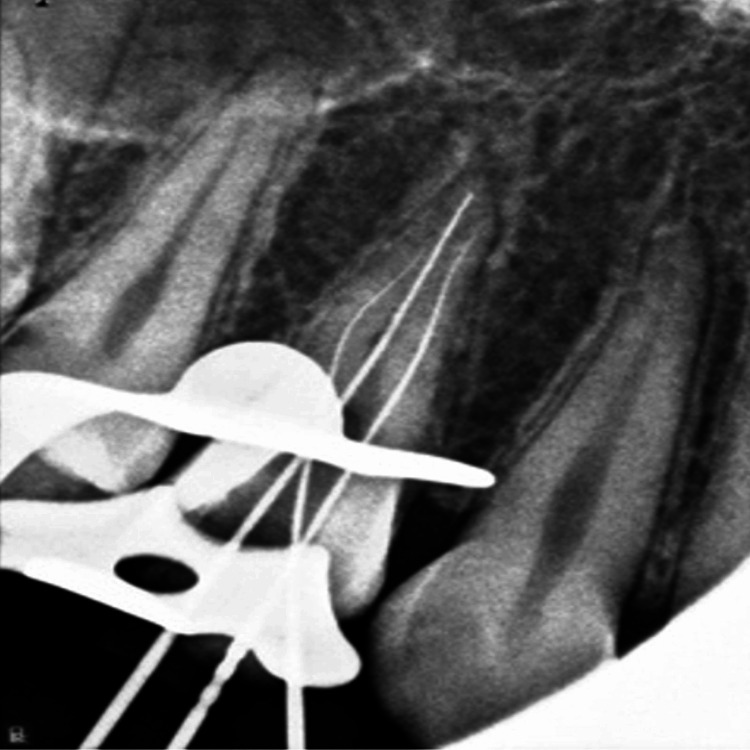
Radiograph of working length

Working length was opted, by keeping the instrument 0.5 mm short of the radiographic apex (Figure 3).

**Figure 4:**
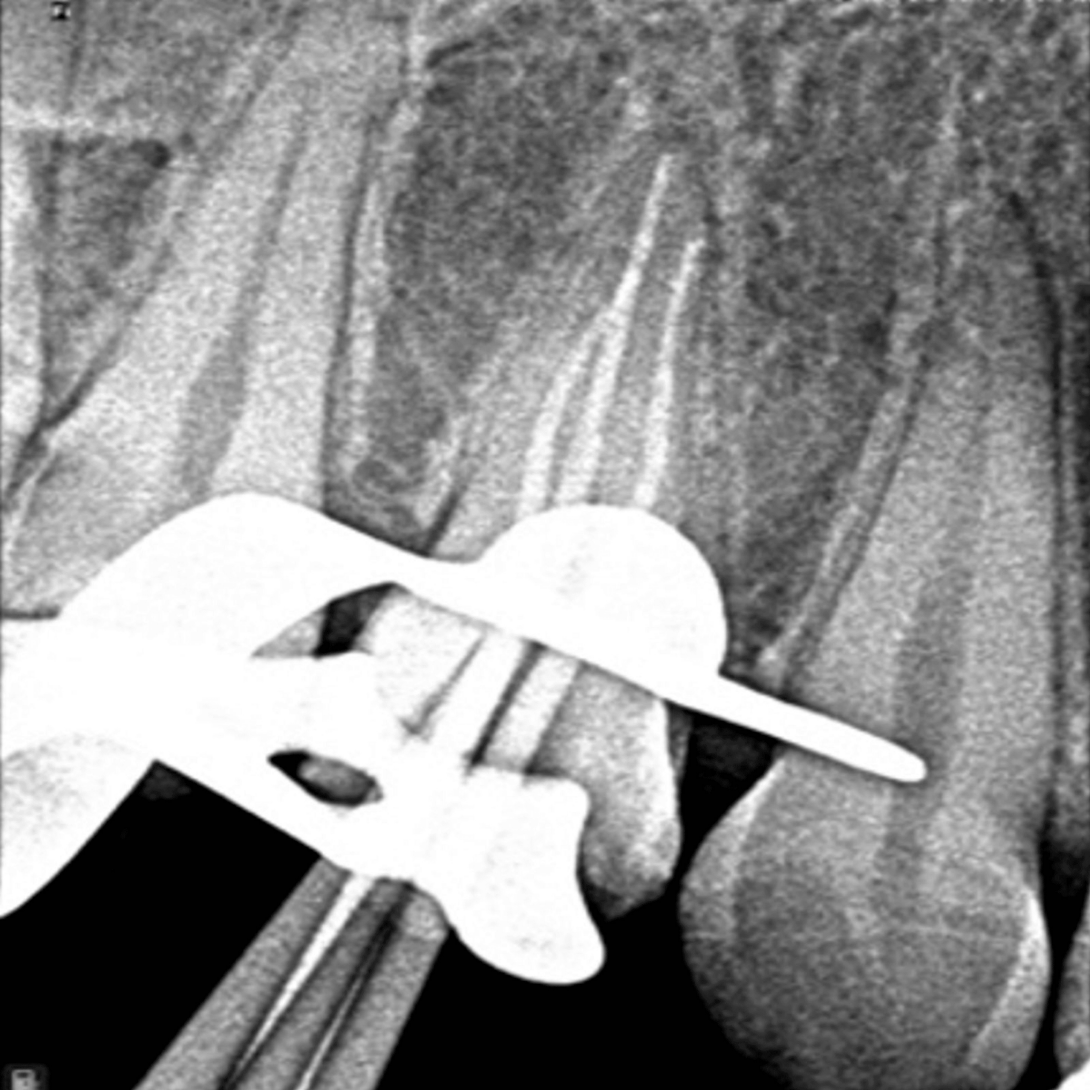
Radiograph of master cone selection

Following the determination of the working length, a hand file up to 15 k-file was used to generate the initial glide approach. Biomechanical preparation was done using Dentsply ProTaper Gold up to F1 (Dentsply ProTaper Gold system, Charlotte, NC) using 3% sodium hypochlorite (PRIME Dental Products Pvt Ltd, Thane, India) and 15% EDTA paste (PRIME Dental Products Pvt Ltd, Thane, India) for debridement. A master cone was selected, and a radiograph was taken for confirmation (Figure 4).

**Figure 5:**
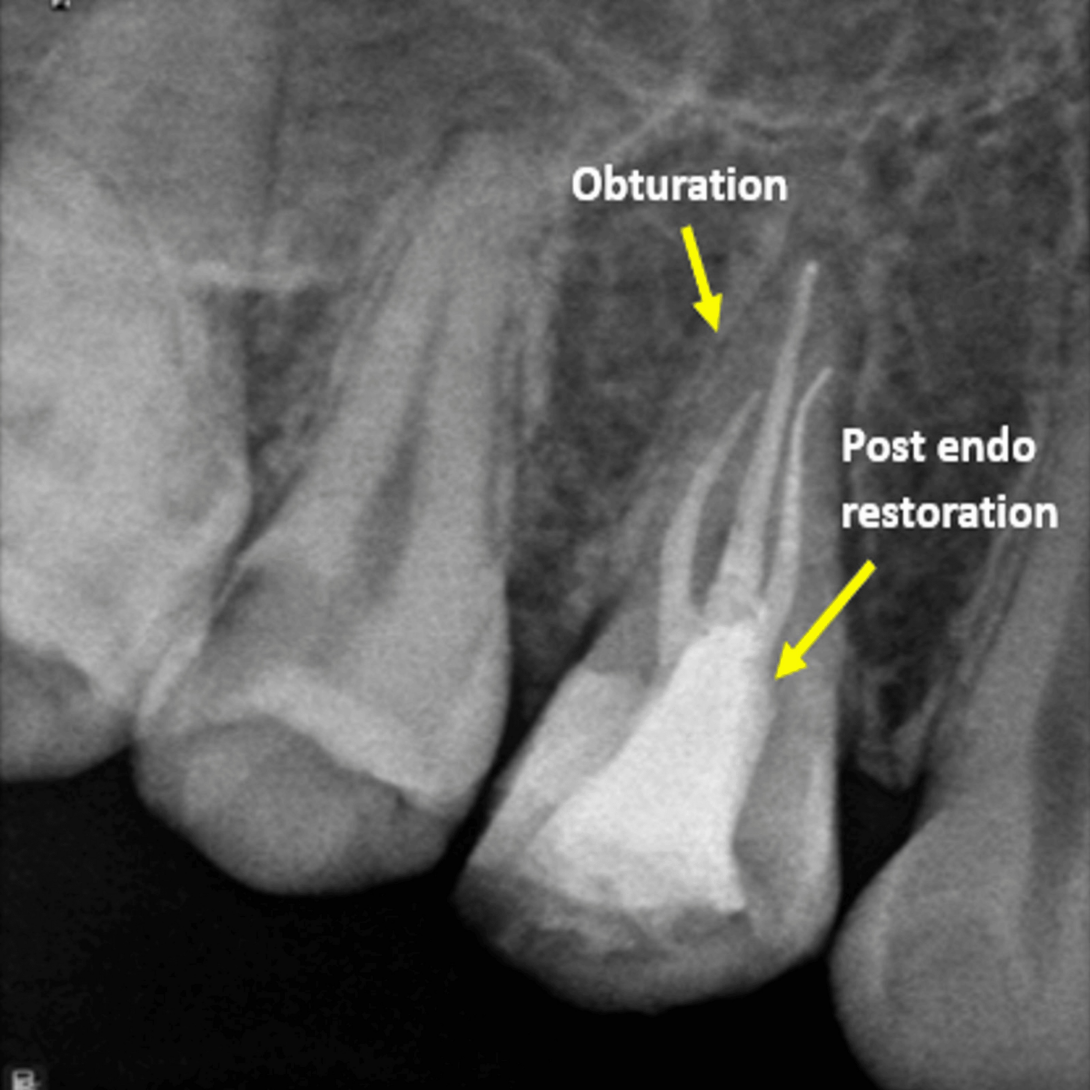
Obturation radiograph

Obturation was done using a single cone technique using AH Plus sealer (Dentsply DeTrey GmbH, Konstanz, Germany), followed by temporary restoration using Cavit (3M ESPE). After seven days, the patient was recalled for follow-up, which revealed no symptoms with the concerned tooth after which post-endodontic restoration was done using Composite (3M ESPE) (Figure 5).

## Discussion

The effectiveness of endodontic treatment depends on complete canal root canal debridement and obturation. Treatment failure may occur if a canal is missed [6]. As a result, clinicians need to be skeptical of both the characteristics of typical root canal features and any potential deviations [6]. The canal and root morphology of the maxillary first premolar vary greatly; it often includes two foramina and distinct canals in 72% of cases [7,8]. According to studies, three canals are present in up to 6% of these teeth [2,7,9]. It can frequently be challenging to locate all three canals in a maxillary bicuspid on preoperative radiographs [4].

During endodontic treatment, anatomical differences can be detected using magnifying loupes and radiographs taken from various angles [10]. Since the generated images are two-dimensional, they might not offer all the information about the canals. Since cone-beam computed tomography (CBCT) was developed and used in endodontic therapy, it is now easy to find root canal orifices that might be left out even using periapical radiographs acquired from different angulations [11]. Additionally, one can effectively locate additional canal orifices by carefully analyzing the floor of an ideally shaped endodontic access cavity and preparing it accordingly [10].

With the help of the right k-file, a thorough tactile examination of the buccal wall was essential in this case to identify the extra buccal canal that was completed using a 10 k-file. To achieve a substantial hermetic seal, special attention was also given to the canal debridement and obturation of the root canals after they had been identified [4]. The optimal conditions for locating canal orifices are dry pulp floors and adequate lighting. Using a microscope or loupes to magnify objects is typically thought to be useful [3,12].

According to Sieraski et al., if the mesial-distal width of the mid-root image appears equal to or greater than the mesial-distal width of the crown image, then the tooth most likely has three roots [13].

Limitations

This study has a limitation that should be acknowledged: the absence of CBCT imaging, which would have allowed for the identification of three distinct canals in premolars. However, a radiovisiography (RVG) revealed the presence of three canals in the tooth of concern, so we did not proceed with CBCT imaging.

## Conclusions

The existence of a second buccal canal and root in a maxillary first bicuspid is an unusual anatomical variation. To treat infections and their accompanying symptoms, endodontists should always take into account the potential for an unusually high number of roots and canals. Advanced devices such as CBCT and microscopes are suggested to treat teeth with intricate root canal anatomy. The accuracy required to manage complex cases is guaranteed, while the final success rate of root canal therapy is increased. Maintaining the normal functioning of the oral environment requires the prompt diagnosis and treatment of the teeth exhibiting these abnormalities. In addition to providing a useful reference for future cases that may be comparable, this example contributes to understanding the anatomical variations and treatment planning of the maxillary first premolar.

## References

[1] Ferreira CM, de Moraes IG, Bernardineli N (2000). Three-rooted maxillary second premolar. J Endod.

[2] (2017). Harty's endodontics in clinical practice. https://books.google.com/books?hl=en&lr=&id=h0PUDAAAQBAJ&oi=fnd&pg=PP1&dq=hartys+endodontics+in+clinical+practice&ots=D0BGzm_B2L&sig=DkaKDahTzBIoidrSbOlueSFUUuo.

[3] Bandewr AA, Majed AH, Shatha AF, Fanan AS, Reem A (2010). Maxillary first premolar with three canals: case report. Smile Dent J.

[4] Sulaiman AO, Dosumu OO, Amedari M (2013). Maxillary first premolar with three root canals: a case report. Ann Ib Postgrad Med.

[5] Kartal N, Özçelik B, Cimilli H (1998). Root canal morphology of maxillary premolars. J Endod.

[6] Yeh CS, Wong WB, Kan WY, Tu MG (2017). Root canal treatment of a three-rooted maxillary second premolar. J Dent Sci.

[7] Carns EJ, Skidmore AE (1973). Configurations and deviations of root canals of maxillary first premolars. Oral Surg Oral Med Oral Pathol.

[8] Dadresanfar B, Khalilak Z, Shahmirzadi S (2009). Endodontic treatment of a maxillary first premolar with type IV buccal root canal: a case report. Iran Endod J.

[9] Pécora JD, Saquy PC, Sousa Neto MD, Woelfel JB (1992). Root form and canal anatomy of maxillary first premolars. Braz Dent J.

[10] Ugur Z, Akpinar KE, Altunbas D (2017). Maxillary first premolars with three root canals: two case reports. J Istanb Univ Fac Dent.

[11] Kakkar P, Singh A (2012). Maxillary first molar with three mesiobuccal canals confirmed with spiral computer tomography. J Clin Exp Dent.

[12] Trope M, Elfenbein L, Tronstad L (1986). Mandibular premolars with more than one root canal in different race groups. J Endod.

[13] Sieraski SM, Taylor GN, Kohn RA (1989). Identification and endodontic management of three-canalled maxillary premolars. J Endod.

